# Assessing Vaccine Hesitancy among Healthcare Workers: A Cross-Sectional Study at an Italian Paediatric Hospital and the Development of a Healthcare Worker’s Vaccination Compliance Index

**DOI:** 10.3390/vaccines7040201

**Published:** 2019-11-29

**Authors:** Sonia Paoli, Chiara Lorini, Francesco Puggelli, Antonino Sala, Maddalena Grazzini, Diana Paolini, Paolo Bonanni, Guglielmo Bonaccorsi

**Affiliations:** 1 Department of Health Science, University of Florence, Viale GB Morgagni 48, 50134 Florence, Italy; chiara.lorini@unifi.it (C.L.); f.puggelli@meyer.it (F.P.); maddalena.grazzini@unifi.it (M.G.); diana.paolini@unifi.it (D.P.); paolo.bonanni@unifi.it (P.B.); guglielmo.bonaccorsi@unifi.it (G.B.); 2 Management Department, Meyer Children’s University Hospital, Viale Gaetano Pieraccini 24, 50139 Florence, Italy; antonino.sala@meyer.it

**Keywords:** vaccine hesitancy, Healthcare workers (HCWs), Vaccine Confidence Index, Italy, paediatrics

## Abstract

Healthcare workers (HCWs) in paediatric hospitals are an important source of advice on vaccinations, but vaccine hesitancy can affect even these professionals. The aim of this study is to assess this phenomenon, measuring it by means of a scoring system. A survey was conducted in five departments of an Italian paediatric university hospital of national interest. Vaccination against influenza was considered a behavioral indicator of vaccination uptake. Using the collected data, the healthcare worker’s vaccination compliance index (HVCI) was computed. The results demonstrate statistically significant differences between departments and professional profiles. Nearly 80% of the sample was not immunized against seasonal influenza. According to the HVCI scores, the most hesitant departments are the intensive care unit, emergency room, and oncohematology department, while the most hesitant professional profiles are nurses and auxiliary staff. The score of the unvaccinated is significantly lower than that of the vaccinated, and the same difference was found between those who self-perceive to be skilled versus unskilled. The HVCI score was statistically verified as a predictive parameter to assess vaccination against seasonal influenza. By means of strategic training policies, both HVCI and perceived skills could be improved, suggesting that hospital management should draw a complex intervention program to fight against hesitancy.

## 1. Introduction

### 1.1. The Vaccine Hesitancy Background

Since the first discoveries of Edward Jenner in 1796 [1], vaccinations have represented a revolution in the field of preventive medicine; however, in spite of the results obtained and the unanimous positions of scientists, in recent years, public health services have been fighting against a growing skepticism towards vaccines. This worldwide problem, known as “vaccine hesitancy”, includes behaviors ranging from a simple tendency to postpone certain vaccinations, to the expression of more or less strong doubts about them, and even a complete refusal to receive of any vaccine shot. In 2015, the Strategic Advisory Group of Experts (SAGE) of the WHO attempted to give a tighter explanation for this multifocal phenomenon, and outlined some basic elements in the so-called complacency, convenience, and confidence model (“3 C” model) [2], where confidence is defined as believing in the efficacy and safety of vaccinations, complacency means having a low perception of risk and the need for vaccinations, and convenience is the perception of the quality, costs, and availability of public health services. Betsch [3] later introduced a fourth “C”, calculating, to describe weighing the pros and cons relating to vaccination. These and other elements define vaccine hesitancy. For this reason, it is not simple to give this phenomenon a proper definition and contextualization, as it can vary according to the social, political, and cultural context of a country or a specific geographical area. According to a 2016 global study on vaccine confidence in collaboration with the Win/Gallup and the Vaccine Confidence Project, over 20% of those surveyed had low confidence in vaccine safety and effectiveness, and 15% did not feel vaccines were important. Azerbaijan, Russia, and Italy reported the most skepticism around vaccine importance, while France reported a global confidence issue [4]; in subsequent studies other European countries also expressed skepticism [5]. Regarding healthcare workers (HCWs), recent studies in Europe have highlighted other problems. In addition to not feeling the moral responsibility to get vaccinated, HCWs are badly informed on the issue and admit that the health centers and hospitals do not actively promote vaccinations [6].

For these and other reasons, among HCWs, flu vaccination rates are still unacceptably low, increasing the perception that a mandate for HCW is needed [7], not only for flu but also for all the preventable infectious diseases that could be a threat to patients, such as Hepatitis B (the vaccine of which has been mandatory in Italy since 1991 and thousands of citizens could have missed it, highlighting the potential need for a control strategy).

### 1.2. Psychological Reactance and Hesitancy Phenomenon

Some years ago, social psychology introduced the concept of “psychological reactance” [8,9], a form of rebellion against imposed rules that the population perceives as restrictive and opposite to principles of self-determination; this could be a further component in the vaccine hesitancy mosaic. In fact, many accusations against vaccinations and their policies are directed at the concept of mandates, perceived as the result of economic agreements between pharmaceutical companies and compliant governments. For example, in 2017, the Italian government, after the drop in vaccination coverage [10,11], approved the “Lorenzin decree” (named after the minister of health, who reintroduced or extended the obligation for anti-poliomyelitis, anti-diphtheria, anti-tetanus, anti-hepatitis B, anti-pertussis, anti-haemophilus influenza b, anti-measles, anti-rubella, anti-mumps, anti-chickenpox vaccinations) [12], but this worsened the clash between the healthcare system and the general population, who perceived this choice as a favor to pharmaceutical companies. In this regard, some studies have demonstrated that making selected vaccines compulsory can produce detrimental effects on the vaccination programs (especially among those who are already hesitant), bringing about a decrease in the uptake of voluntary vaccinations [13].

The population’s protest against vaccination science and policy are usually expressed on the Internet and via social networks, through the dissemination of messages with strongly persuasive features, often linked to fake discoveries like the false correlation between vaccines and autism [14]. These kinds of messages have multiplied exponentially and are gathered by an increasing number of users who choose the Internet as their primary source of information [15,16].

These persuasive messages can be elaborated by the user through the search for additional information, thereby resisting the message itself, or in a superficial way, choosing elements that are not part of the core of the message, but evoke particular emotions. Many variables can change the way we react to a persuasive message: The perception of a hazard and the possibility of avoiding it, the motivation of the subject, one’s basic cultural level, and the ability to access the right information and to process it effectively. Moreover, the use of social pressure coming from one’s contacts, (the so-called “ingroup”), is important for the negative or positive influence that can be exerted on the subject’s opinions, based on a sense of identification within that group. A cognitive-behavioral model (PRISM) explains these concepts well [17].

### 1.3. The Survey

A cross-sectional study was conducted in the unique environment of a paediatric university hospital, where a patient is not merely subjected to vaccination but is also completely dependent on his or her parents; therefore, the staff has an even more important role and must proactively direct the parents towards the appropriate choices regarding vaccination.

From this perspective, our study aimed at exploring the following endpoints.

The First endpoint is to produce a scoring system of hesitancy by means of a healthcare worker’s vaccine compliance index (HVCI) to be specifically applied to the population of the sample (HCWs who attended the selected wards described below).

Secondary endpoints aim at verifying the following hypotheses (HP):

*HP 1*: There are differences in the HVCI score between hospital departments.

*HP 2*: There are differences in the HVCI score between professional categories.

*HP 3*: There are differences in the HVCI score between those who received and who did not receive the flu shot.

*HP 4:* The HVCI score of those who are assessed to be skilled in vaccination is greater than the HVCI score of those who are not.

*HP 5*: The HVCI scores among subjects in the “influential” ingroup is lower than the HVCI score of those who belong to the “not influential” ingroup.

*HP 6:* The HVCI score is a statistical predictor of flu vaccination occurrence, as a net of other variables (age, sex, health status, current diseases, and perceived skills in vaccination themes).

## 2. Materials and Methods

The study was carried out from June to August 2018 using an anonymous paper questionnaire taken by HCWs from the intensive care unit, the health management, diagnostics, and oncohematology departments, and the emergency room. These departments have different complexities linked to the characteristics of the patients, and employ different types of professionalism, thus offering a potential global vision of this phenomenon in a hospital setting. The health management department was chosen for the specific key function of the control and the prevention of infectious diseases within the hospital and for the strategic choices that could be made in this field. Assessing a hesitancy index is also crucial in the oncohematology and intensive care units, since both of them host particularly fragile patients susceptible to the worst consequences of infectious diseases. The emergency room and diagnostics department are a first contact zone with a high turnover of external patients and health emergencies. HCWs working in these contexts manage patients whose diagnoses are not yet known, thereby exposing them to a higher risk of meeting infectious agents.

The professional figures interviewed were doctors, nurses, healthcare assistants, and lab technicians. No age limits were set.

The survey tool (questionnaire) was built as follows:

(1) Generalities, where the HCWs provided information about their department, professional profile, age, gender, and health status;

(2) Self-assessment on expertise regarding vaccinations. A self-judgment was requested (“excellent”, “good”, “discrete”, “poor”); subsequently, a “skilled” subgroup (including those who gave “excellent” and “good” judgments to their own competences) and an “unskilled” one (including those who gave the “poor” and “discrete” judgments) were identified;

(3) Attitude towards flu vaccinations, including items investigating the occurrence (or absence) of vaccination against influenza; this was considered to be an indicator of the attitudes related to vaccination. The motivation to vaccinate or not was also requested;

(4) Confidence, compliance, and risk perception were analyzed via 19 statements (see Table 1) in the form of a Likert 4-point scale, where “1” corresponds to “I do not agree” and “4” corresponds to “I totally agree”;

Subsequently, a healthcare worker’s vaccine compliance index was built, according to the body of European literature. The questions of the survey were related to other scientific papers [15,16,17] that investigated beliefs and attitudes toward vaccinations among HCWs in Italy. Another body of questions was related to the HproImmune Project [6], the Vaccine Confidence Project [18,19,20], and a recent Italian research [21]. The whole survey was fulfilled by 10 HCWs involved in the pretesting phase in order to assess the comprehensibility and the possible presence of errors. Ten statements were taken from the pool of statements in Section 4 of the survey, according to the most commonly used keywords related to vaccines found on web search engines, in order to collect half of the statements into an “A” group (corresponding to each assertion where the higher the Likert score, the better the propensity towards vaccines), and the other half into a “B” group (matching each assertion where the higher the Likert score, the lower the propensity). The items selected were numbers 1, 3, 5, 6, 8, 10, 11, 12, 14, 18 in order to cover the main aspects of hesitancy (confidence, complacency, convenience) and to produce a simple, short index. The other statements were not considered suitable for index calculation. In fact, some of them were related to the concept of being mandatory (4, 7, 16, 17, 19), which is linked to the concept of self-determination; these two elements can be investigated separately. Other items were very similar to the ones already computed in the index (2), or could lead to misinterpretations (9, 13, 15).

The healthcare worker’s vaccine compliance index was calculated as follows:


HVCI = [Σ (A1 + A2..An)/Σ (A)]/[Σ (B1 + B2..Bn)/Σ (B)].

(5) The ingroup role and affective responses. We investigated complacency among the ingroup of the sample with a set of multiple choice questions regarding what HCWs felt when they learned about the drop-in vaccination coverage and what their colleagues and contacts think about the measles epidemic and the need to get vaccinated.

The last two questions asked the sample if they had ever heard or seen their contacts or colleagues talking about serious vaccine reactions. Considering the answers “yes” or “no” to these last two questions, we divided the sample into two subgroups, the “influential ingroup” (answered “yes” to both) and the “not influential ingroup” (answered “no” to both). Mixed answers (yes/no) were not considered.

The data were collected, computerized, and subsequently processed using IBM SPSS Statistics 25. The associations between individual responses and specific professions or departments were assessed by a Chi-Square test. The normality of the HVCI score distribution was evaluated using a Kolmogorov–Smirnov test. The associations between the score and the variables listed in Section 4 were evaluated by ANOVA, Student’s *t* test or corresponding nonparametric tests. The predictive role of HVCI and other variables with respect to the uptake of flu shots in 2017–2018 (sex, age, department, professional profile, health status, occurring diseases, and perceived skills) were tested with a multivariate logistic regression analysis, using a backward stepwise procedure, and the fitting of the final model was proven with a Hosmer–Lemeshow test. For all analyses, a *p* level of 0.05 was considered statistically significant

Ethics Committee: The study was approved by the Paediatric Ethics Committee—Tuscany Region (Protocol VHOS2616, approved on 28/05/2018) and was conducted according to the principles expressed in the Helsinki Declaratio.

## 3. Results

The number of collected questionnaires is 108, equal to 31% of the HCWs employed in the investigated departments.

Table 2 reports the generalities of the sample. As shown in the table, statistically significant differences emerged between departments if we consider the professional profiles that gave more answers. Chronic diseases were also assessed. Among the respondents, 76% had no chronic diseases, 9.3% suffered from an autoimmune disease, 7.4% from a respiratory disease, 4.6% from a cardiovascular disease, 2% from diabetes, and 1% from a renal disease, without statistically significant differences between hospital departments.

Table 3 shows the percentage of those who participated and filled in the questionnaire and those who did not.

As shown in Table 4, approximately 17% of the respondents considered themselves to be poorly competent in understanding vaccinations. This was especially true for the 24–35 age group (*p* < 0.01), while no statistical differences were found between departments. Forty-seven percent of physicians affirmed to be highly skilled, compared to 38% those in other professions. Nevertheless, no statistical difference was found.

Nearly 80% of the sample was not vaccinated against influenza in 2017–2018. These data are not affected by the statement of suffering from chronic diseases. Eighty-three percent of women did not get vaccinated compared to 71% of men (*p* < 0.05). The oncohematology department had the highest percentage of unvaccinated workers (86%), and diagnostics had the lowest (76%), although these differences are not statistically significant. Flu vaccine uptake significantly (*p* < 0.001) differs by occupational categories. The highest unvaccinated percentage belongs to the auxiliary staff (100%) and the lowest to physicians (63%). Those who received the vaccination answered that they want to be immunized in order to protect patients (34%) and consider the vaccine safe and effective (30%), while the unvaccinated respondents answered that they do not consider themselves at risk (25%), reported never getting sick (21%), and think that influenza is not a serious disease (31%). Among the other strongly recommended vaccinations for healthcare workers, hepatitis B was also investigated. Almost all of the sample had a hepatitis B vaccination, but two nurses did not. These nurses affirmed that they did not consider it relevant.

Table 5 shows the attitudes and opinions about vaccinations that emerge from the Likert scale score. The P-value scores show some differences between departments. As can be seen, the intensive care unit, oncohematology department, and the emergency room scored more in some of the statements representing aspects of hesitancy; they also scored lower in some areas that less represent them.

Table 6 shows that the differences in hesitancy are much more relevant if we draw a comparison between job classes. As can be seen, nurses and auxiliary staff scored higher in some of the statements that most represent them; they also scored lower in some areas that less represent them.

Subsequently, the healthcare worker’s vaccine compliance index was calculated for each single questionnaire, omitting the ones (n = 2) with missing values in the numerator or the denominator of the index. The minimal value of the HVCI of the sample is 0.462, and the maximum value is 4 (mean = 2.104, median = 2, average = 2). We calculated the average HVCI of the subgroups and verified the hypothesis shown in Table 7.

The results of the hypothesis testing phase are reported as follows.

*(HP1)* There is a significant difference (*p* < 0.05) in the HVCI score between the hospital departments. The highest score was obtained from the management, and the lowest was obtained from the intensive care unit.

*(HP2)* Professional classes present different HVCI scores (*p* < 0.001). The highest was obtained by physicians, and the lowest was obtained by the auxiliary staff.

*(HP3)* Vaccinated and unvaccinated against the flu (year 2017–2018) subjects presented significantly different HVCI scores (*p* < 0.001, higher in vaccinated subgroup).

*(HP4)* Skilled HCWs presented a significantly higher HVCI score then unskilled ones (*p* < 0.05).

Subsequently, the HCWs were asked about their feelings regarding the decrease in vaccination coverage. Sixty-seven percent experienced anger, 19% were afraid and fearful for their contacts, while 8% took an avoidant attitude and did not care about the problem.

For 45% of the sample, the colleagues had very mixed opinions about the need to get vaccinated after the measles epidemic: 6.5% claimed that their colleagues do not consider this epidemic a real emergency. At the same time, 26% stated they had learned about serious vaccine reactions from their colleagues; of these, more than half (53.6%) belong to the intensive care unit, the area with the lowest HVCI score (*p* < 0.05).

For 72% of the HCWs, contacts (excluding colleagues) have conflicting thoughts on the need for vaccines; 17.6% stated that the impressions of their contacts about vaccinations are negative, and 32% said that they heard about severe vaccine reactions from their patients (or their parents).

These results have led us to believe that there could be a correlation between what HCWs see or hear, both in the workplace and outside (in their ingroups) and their attitudes and opinions toward vaccinations. This led us to consider Hypothesis 5. 

*(HP5)* The average HVCI differs among HCWs with an influential ingroup, with p-values at the limits of statistical significance, based on the HVCI scores of those who do not have an influential ingroup.

*(HP6)* The likelihood that some variables of the model can predict the occurrence of the flu vaccination was tested. A multivariate logistic regression model was performed, including the flu vaccination as a dependent variable (1 = having received the vaccine shot in 2017–2018; 0 = not having received the vaccine shot in 2017–2018), and sex, age, department, professional profile, health status, occurring diseases, perceived skills, and HVCI score were used as independent variables. Using a backward stepwise procedure, the variables that were found unrelated were deleted and the final model obtained shows that the possible occurrence of vaccination against flu is influenced by following variables (as shown in Table 8): sex, perceived skills, and healthcare worker’s vaccine compliance index score. HVCI gave the best result with *p* = 0.001.

The fitting of the model was assessed: the Hosmer–Lemeshow test showed a *p* > 0.05 (*p* = 0.27).

## 4. Discussion

### 4.1. Summary of Findings

This research shows the relevant hesitancy issues regarding the perception of the risks, needs, benefits, and efficacy of vaccinations. The department that turned out to be most doubtful was the intensive care unit, the emergency room, and the oncohematology department, while the HCWs that turned out to be most doubtful were nurses and auxiliary staff. The coverage of the seasonal influenza vaccination is low, and this fact is similar in other European studies, in which the reported mean coverage is less than 20% [22,23]. Physicians get vaccinated more than those in other categories and also had a higher HVCI score. This score, the healthcare worker’s vaccine compliance index, was demonstrated to be a predictor of the occurrence of the flu vaccination, the net of the other variables that were also good predictors (sex and perceived skills).

An important element that emerged from this survey is that there is a perceived shortcoming in the basic cultural training of HCWs, underlined by a low judgment of their own self-competencies in the vaccination field, a feature already found in other studies carried out in other European countries [5]. Those who are vaccinated perceive themselves to be more knowledgeable about vaccinations and show a willingness to offer protection both for themselves and for their contacts; they also consider vaccines to be effective and influenza as a potentially serious threat. HCWs who do not receive the flu shot do not feel that they are at risk, so they pay more attention to individual protection. They also do not consider the flu to be a dangerous disease or think that they will get sick regardless, thus doubting the effectiveness of the vaccine. These attitudes are in line with many surveys conducted at the national and European level [22,23,24,25,26,27,28].

### 4.2. Strengths and Limitations

HVCI is a predictor of the flu vaccination for 2017–2018: This result should be carefully considered because the HVCI could be improved with strategic training policies, as well as improving perceived skills, which are another good predictor.

As a limitation, only 31% of the possible population took part in the survey. Table 3 shows the percentage of responders for every department and professional class. This limitation must be considered in terms of the possible presence of distorted elements in the data analysis (e.g., a selection of those who participated), and as a limit in the generalization of the results. The causes for the poor response are unknown but can be attributed to the length of the questionnaire, to the collecting method (five locked boxes, one for each ward), and to a possible “avoidance phenomenon” due to the high frequency with which the staff of this hospital is routinely subjected to surveys on many different themes.

### 4.3. Implication and Future Work

A relevant fact that emerged from this survey is that besides HVCI and perceived skills, sex is also a predictor of flu vaccination; the percentage of women vaccinated against the flu was significantly lower than the percentage of men vaccinated. This result recalls similar findings in other Europeans studies [5,6]. There is the possibility of a “gender gap” and this requires more attention and future investigations.

Physicians are the most prone to engage in correct vaccination practices and would also be more amenable to a possible extension of mandatory vaccinations, both for health workers and for public employees. The obligation for HCWs is a theme that needs to be investigated further, especially in Europe, since it has already been studied outside Europe [29,30] with strongly positive results. In this case, it is appropriate to consider the ethics of this provision and whether it would conflict with freedom of choice and self-determination. Vaccination facilities (such as mobile vaccination vans) or the role models of senior HCWs receiving vaccination are among the strategies that have been observed to improve vaccination uptake rates [30], and could be better applied in Europe. Other studies report that these strategies need to be prolonged over time for the best results, suggesting that hospital management should draw a complex intervention program that includes a variety of coordinated managerial and organizational elements [31]. Combined strategies are more effective than isolated approaches [23]. This must be considered a beneficial endeavor not only for HCWs’ (and patients) health but also for the proper functioning of the hospital. Indeed, it has been demonstrated that increasing flu vaccine coverage can decrease sickness-based absence rates [32].

Psychosocial theories state that one’s network of contacts can bring about a change in the attitudes and opinions of the subjects. The data show that in some cases, HCWs have a hesitant ingroup that discusses the negative effects of vaccination and could influence the opinion and attitudes of HCWs. In our research, this phenomenon happened more commonly in departments with lower HVCIs. Nonetheless, more studies are needed to verify whether this fact can be generalizable to other healthcare contexts.

## 5. Conclusions

This survey shows that vaccine hesitancy issues exist and are relevant in the investigated hospital, with substantial differences between departments and professional classes. The departments most affected by this phenomenon are those that exert a predominant role in taking care of critical diseases (the intensive care unit and the oncohematology department) or managing the first contact with patients (the emergency room), namely those who should be more confident about vaccination. HCWs are still among the most trusted influencers about vaccinations. For this reason, finding a skeptical professional could strongly change people’s mind, or reinforce the idea that vaccinations are unsafe, especially among those who already refuse vaccinations. The need to strengthen trust in vaccines goes with the need to improve communication skills with patients. HCWs have the duty to inform people about vaccinations and the risks that result from poor coverage, but these professionals often face a lack of time, are not up to date, and do not feel the need to raise the awareness of these issues. New strategies need to be found in order to improve their knowledge, confidence, and communication skills [33,34,35]. As expected, the propensity towards good vaccination policy differs significantly between physicians, nurses, and auxiliary staff, and this can be related partially to their cultural level, differences between their university courses, and the availability of refresher courses; this could also be related to the social backgrounds of these professionals. These element could be a starting point to build a multilevel strategy of training.

In this regard, the results from the HVCI model demonstrate that being a physician leads to a more conscious, more confident, and more compliant way of thinking about vaccinations. This could be because of their deeper knowledge of the composition and function of a vaccine (even if only self-perceived), or it could depend on their greater responsibility towards the patient and their moral duty not to be harmful. Nurses and auxiliary staff could feel less responsibility or could not be up to date in their vaccination knowledge. Scientific studies on the link between knowledge and some aspects of this hesitancy do not always show direct proportionality, suggesting that hesitancy is a complex phenomenon that does not depend only on a good knowledge of vaccinations [36].

The model also shows that a high HVCI score leads to a better propensity to get vaccinated against seasonal flu. A further statistical analysis showed that the flu vaccination can be predicted by the HVCI score and self-perceived knowledge. By all accounts, despite vaccine hesitancy being a multifocal phenomenon, the culture and (most importantly) the homogeneity of attitudes and opinions of health personnel could be strong weapons against ideological drift in the health field.

## Figures and Tables

**Table 1:** Statements from Section 4 of the survey (confidence, compliance, and risk perception).

1. I think that every paediatric vaccine is needed
2. In the case of infrequent diseases, you can defer a scheduled vaccine
3. In some circumstances, if the child is healthy, he or she can get along without the vaccine
4. I agree with the new vaccination policy program (Lorenzin decree) ^a^
5. I do not feel comfortable when I recommend vaccination for a weak child
6. It is better if a child gets immunized in a more natural way, rather than using a vaccine
7. Every family should have the right to choose whether their baby should be vaccinated
8. Being up to date on vaccination themes is needed in my job
9. Influenza is an overvalued disease
10. My contacts expect me to be up to date on vaccinations
11. I constantly recommend the flu shot to my colleagues
12. I constantly recommend the flu shot for those who are diabetic and suffer from heart disease or renal failure
13. Since the mass media are talking about adverse vaccine reactions, I trust vaccines less than before
14. Some of the side effects of vaccines are kept hidden from us
15. Vaccine excipients have serious side effects
16. There should be more mandatory vaccines for health professionals
17. There should be more mandatory vaccines for those who work with the public
18. I believe that the health surveillance of adverse vaccine reactions is ineffective
19. Everyone should have the right to choose whether or not to get vaccinated

^a^ This question is specific for Italy as it refers to the mandatory program for anti-poliomyelitis, anti-diphtheria, anti-tetanus, anti-hepatitis B, anti-pertussis, anti-haemophilus influenzae b, anti-measles, anti-rubella, anti-mumps, and anti-chickenpox vaccinations. It investigates the feelings toward mandates but will not be considered for the HVCI.

**Table 2:** Generalities of the participating staff by hospital departments. ICU: Intensive care unit; MAN: management; DIAG: diagnostics; EMERG: emergency room; ONC-HEM: oncohematology. PHIS: physicians; NUR: nurses; AUX: auxiliary staff.

GENERALITIES		ICU (%)	ONC-HEM (%)	EMERG (%)	DIAG (%)	MAN (%)	TOTAL	*p*
PROFESSION *	PHIS	8.3	0	28	45	18.2	21	<0.01
	NUR	83.3	85.7	60	15	81.8	71	
	AUX	8.1	14.3	12	40	0	16	
SEX	FEMALE	86.5	85.7	28	65	81.8	73	0.33
	MALE	13.5	14.3	72	35	18.2	34	
AGE	<25	0	0	4	0	0	1	0.22
	26–35	0	50	32	35	27.3	25	
	36–45	29.7	28.6	24	45	9.1	31	
	46–55	32.4	21.4	32	10	45.5	30	
	56–65	37.8	0	8	10	18.2	20	
	>65	0	0	0	0	0	0	
YEARS OF PRACTICE	<5	10.8	35.7	24	25	9.1	21	0.30
	From 6 to 10	32.4	14.3	24	30	18.2	28	
	From 11 to 20	32.4	42.9	20	30	18.2	31	
	From 21 to 30	21.6	7.1	24	5	45.5	21	
	From 31 to 40	2.7	0	8	10	9.1	6	
	>41	0	0	0	0	0	0	

* *p* < 0.05

**Table 3:** Percentage of responders and nonresponders among departments and professional categories. ICU: Intensive care unit; MAN: management; DIAG: diagnostics; EMERG: emergency room; ONC-HEM: oncohematology. PHIS: physicians; NUR: nurses; AUX: auxiliary staff. Please note that the management department has no auxiliary staff.

RESPONDERS	PHIS		NUR		AUX		TOTAL % (Y)	TOTAL % (N)
	Y (%)	N (%)	Y (%)	N (%)	Y (%)	N (%)		
ICU	0.86	10.66	8.93	18.73	0.86	4.32	10.66	33.72
MAN	0.58	0.86	2.59	2.31	0.00	0.00	3.17	3.17
DIAG	2.59	1.73	0.86	0.29	2.31	4.32	5.76	6.34
EMERG	2.02	3.75	4.61	6.92	0.86	0.86	7.49	11.53
ONC-HEM	0.00	4.32	3.46	8.07	0.58	1.73	4.03	14.12
TOTAL (%)	6.05	21.33	20.46	36.31	4.61	11.24	31.12	68.88

**Table 4:** Vaccination themes skills and whether or not influenza vaccination has been carried out, and why. ICU: Intensive care unit; MAN: management; DIAG: diagnostics; EMERG: emergency room; ONC-HEM: oncohematology. PHIS: physicians; NUR: nurses; AUX: auxiliary staff.

			ICU			ONC-HEM			EMERG			DIAG			MAN			TOTAL (N)	*p*
			PHIS (%)	NUR (%)	AUX (%)	PHIS (%)	NUR (%)	AUX (%)	PHIS (%)	NUR (%)	AUX (%)	PHIS (%)	NUR (%)	AUX (%)	PHIS (%)	NUR (%)	AUX (%)		
SKILLS		Excellent	0.00	0.00	0.00	0.00	0.00	0.00	33.33	0.00	0.00	0.00	0.00	0.00	33.33	33.33	0.00	3	0.18
		Good	0.00	29.73	2.70	0.00	8.11	0.00	5.41	18.92	2.70	13.51	2.70	5.41	2.70	8.11	0.00	37	
		Discrete	2.08	31.25	0.00	0.00	14.58	4.17	6.25	8.33	4.17	6.25	2.08	10.42	0.00	10.42	0.00	48	
		Poor	11.76	23.53	11.76	0.00	11.76	0.00	0.00	23.53	0.00	5.88	5.88	5.88	0.00	0.00	0.00	17	
FLU SHOT		Yes	9.09	27.27	0.00	0.00	9.09	0.00	22.73	0.00	0.00	13.64	9.09	0.00	9.09	0.00	0.00	22	0.92
		Not	1.22	28.05	3.66	0.00	12.20	2.44	2.44	17.07	3.66	7.32	1.22	9.76	0.00	10.98	0.00	82	
WHY yes?	I am at risk		20.00	20.00	0.00	0.00	0.00	0.00	20.00	0.00	0.00	20.00	0.00	0.00	20.00	0.00	0.00	5	0.91
	I work in a dangerous department		12.50	25.00	0.00	0.00	12.50	0.00	37.50	0.00	0.00	12.50	0.00	0.00	0.00	0.00	0.00	8	
	I do not want to get sick		0.00	33.33	0.00	0.00	0.00	0.00	33.33	0.00	0.00	33.33	0.00	0.00	0.00	0.00	0.00	3	
	I want to take care of the patients		5.88	35.29	0.00	0.00	11.76	0.00	17.65	0.00	0.00	17.65	5.88	0.00	5.88	0.00	0.00	17	
	Flu can worsen a severe illness		0.00	0.00	0.00	0.00	0.00	0.00	0.00	0.00	0.00	0.00	50.00	0.00	50.00	0.00	0.00	2	
	I often get sick		0.00	0.00	0.00	0.00	0.00	0.00	0.00	0.00	0.00	0.00	0.00	0.00	0.00	0.00	0.00	0	
	I think that the flu shot is safe and effective		6.67	26.67	0.00	0.00	6.67	0.00	20.00	0.00	0.00	20.00	13.33	0.00	6.67	0.00	0.00	15	
WHY not?	I am not at risk		0.00	24.00	0.00	0.00	8.00	0.00	0.00	24.00	0.00	12.00	4.00	20.00	0.00	8.00	0.00	25	0.06
	I do not work in a dangerous department		0.00	0.00	0.00	0.00	0.00	0.00	0.00	0.00	0.00	0.00	0.00	0.00	0.00	100.00	0.00	3	
	I do not have time		0.00	25.00	0.00	0.00	25.00	0.00	0.00	0.00	0.00	50.00	0.00	0.00	0.00	0.00	0.00	4	
	I fear needles		0.00	0.00	0.00	0.00	100.00	0.00	0.00	0.00	0.00	0.00	0.00	0.00	0.00	0.00	0.00	1	
	I do not think that the flu is a dangerous disease		0.00	30.00	10.00	0.00	6.67	6.67	3.33	26.67	0.00	6.67	0.00	6.67	0.00	3.33	0.00	30	
	I never get sick		4.76	19.05	0.00	0.00	14.29	0.00	4.76	19.05	9.52	0.00	0.00	9.52	0.00	19.05	0.00	21	
	When I received the flu shot, I still got sick		0.00	35.71	0.00	0.00	28.57	0.00	0.00	7.14	7.14	7.14	0.00	0.00	0.00	14.29	0.00	14	

**Table 5:** Mean, standard deviation, and median values in the Likert scale related to the statements reported in the table; comparison between departments with significance tests. ICU: Intensive care unit; MAN: management; DIAG: diagnostics; EMERG: emergency room; ONC-HEM: oncohematology.

SECTION 4: DEPARTMENTS	ICU		MAN		DIAG		EMERG		ONC-HEM		*p*
	Mean (± SD)	Median	Mean (± SD)	Median	Mean (± SD)	Median	Mean (± SD)	Median	Mean (± SD)	Median	
1. I think that every paediatric vaccine is needed	3.44 (± 0.61)	3.5	3.63 (± 0.50)	4	3.52 (± 0.66)	4	3.4 (± 0.91)	4	3.35 (± 0.84)	3.5	0.906
2. In the case of infrequent diseases, you can defer a scheduled vaccine	1.75 (± 0.98)	1	1.54 (± 0.69)	1	1.66 (± 0.97)	1	1.96 (± 0.96)	2	2 (± 1.04)	2	0.594
3. In some circumstances, if the child is healthy, he or she can get along without the vaccine	1.11 (± 0.32)	1	1.27 (± 0.71)	1	1.16 (± 0.38)	1	1.69 (± 1.12)	1	1.46 (± 0.78)	1	0.066
4. I agree with the new vaccination policy program (Lorenzin’s Decree)	3.21 (± 0.98)	4	3.27 (± 1.19)	4	3.5 (± 0.92)	4	3 (± 1.04)	3	2.78 (± 1.12)	3	0.214
5. I do not feel comfortable when I recommend vaccination for a weak child *	2.05 (± 0.94)	2	1.27 (± 0.47)	1	1.27 (± 0.57)	1	1.84 (± 1.01)	1.5	1.23 (± 0.44)	1	0.002
6. It is better if a child gets immunized in a more natural way, rather than using a vaccine	1.37 (± 0.79)	1	1.36 (± 0.92)	1	1.27 (± 0.67)	1	1.5 (± 0.76)	1	1.64 (± 0.84)	1	0.442
7. Every family should have the right to choose whether their baby should to be vaccinated	1.56 (± 0.90)	1	1.45 (± 0.82)	1	1.27 (± 0.46)	1	1.92 (± 1.04)	2	1.64 (± 0.84)	1.5	0.257
8. Being up to date on vaccination themes is needed in my job	3.29 (± 0.73)	3	3.54 (± 0.69)	4	3.63 (± 0.63)	4	3.65 (± 0.56)	4	3.64 (± 0.50)	4	0.135
9. Influenza is an overvalued disease	2.32 (± 0.92)	2	2.27 (± 1.10)	2	2 (± 0.88)	2	2.34 (± 1.02)	2.5	2.57 (± 1.02)	2.5	0.559
10. My contacts expect me to be up to date on vaccinations	2.72 (± 0.82)	3	2.9 (± 0.94)	3	3.16 (± 0.86)	3	2.96 (± 0.87)	3	2.71 (± 1.14)	2.5	0.385
11. I constantly recommend the flu shot to my colleagues	1.91 (± 1.01)	2	2.45 (± 1.21)	2	2.57 (± 1.30)	3	1.73 (± 0.92)	1.5	1.92 (± 1.07)	2	0.140
12. I constantly recommend the flu shot for those who are diabetic and have heart disease or renal failure	2.97 (± 1.01)	3	3.36 (± 0.67)	3	3.15 (± 1.12)	4	3.12 (± 1.08)	3.5	3.28 (± 0.73)	3	0.806
13. Since the mass media talk about adverse vaccine reactions, I trust vaccines less than before	1.67 (± 0.94)	1	1.27 (± 0.65	1	1.26 (± 0.56)	1	1.53 (± 0.95)	1	1.42 (± 0.65)	1	0.383
14. Some of the side effects of vaccines are kept hidden from us	1.72 (± 0.96)	1	1.18 (± 0.40)	1	1.73 (± 1.10)	1	1.38 (± 0.70	1	2.21 (± 1.23)	2	0.056
15. Vaccine excipients have serious side effects	1.75 (± 0.65)	2	1.54 (± 0.69)	1	1.77 (± 0.73)	2	1.84 (± 0.92)	2	1.76 (± 0.83)	2	0.903
16. There should be more mandatory vaccines for health professionals	2.67 (± 1.04)	3	2.63 (± 1.12)	3	3 (± 1.03)	3	2.3 (± 1.29)	2	2.3 (± 1.11)	2	0.302
17. There should be more mandatory vaccines for those who work with the public	2.7 (± 1.02)	3	2.54 (± 1.04)	3	3.11 (± 1.06)	3	2.3 (± 1.23)	2	2.38 (± 1.12)	3	0.185
18. I believe that the health surveillance of adverse vaccine reactions is ineffective	2.13 (± 0.92)	2	1.45 (± 0.69)	1	1.77 (± 0.73)	2	2.23 (± 0.91)	2	1.92 (± 1.04)	2	0.096
19. Everyone should have the right to choose whether or not to get vaccinated *	2.1 (± 1.15)	2	1.54 (± 0.69)	1	1.61 (± 1.04)	1	2.57 (± 1.21)	3	2 (± 0.89)	2	0.031

* *p* < 0.05

**Table 6:** The mean and median values of the Likert scale related to the statements reported in the table; comparison between job category with significance tests. PHIS: Physicians; NUR: nurses; AUX: auxiliary staff.

SECTION 4: PROFESSIONAL CLASSES	PHIS		NUR		AUX		*p*
	Mean (± SD)	Median	Mean (± SD)	Median	Mean (± SD)	Median	
1. I think that every paediatric vaccine is needed	3.71 (± 0.46)	4	3.38 (± 0.75)	3.5	3.33 (± 0.78)	3.5	0.159
2. In the case of infrequent diseases, you can defer a scheduled vaccine *	1.23 (± 0.44)	1	1.86 (± 0.97)	2	2.42 (± 1.02)	2.5	0.001
3. In some circumstances, if the child is healthy, he or she can get along without the vaccine	1.14 (± 0.48)	1	1.36 (± 0.77)	1	1.5 (± 0.67)	1	0.135
4. I agree with the new vaccination policy program (DDL Lorenzin) *	3.47 (± 0.87)	4	3.15 (± 1.05)	4	2.64 (± 1.01)	3	0.034
5. I do not feel comfortable when I recommend vaccination for a weak child *	1.09 (± 0.30)	1	1.82 (± 0.96)	1.5	2 (± 0.68)	2	<0.001
6. It is better if a child gets immunized in a more natural way, rather than using a vaccine *	1 (± 0)	1	1.42 (± 0.77)	1	2.14 (± 0.95)	2.5	<0.001
7. Every family should have the right to choose whether their baby should be vaccinated *	1.28 (± 0.64)	1	1.61 (± 0.93)	1	2.07 (± 0.73)	2	0.005
8. Being up to date on vaccination themes is needed in my job	3.57 (± 0.75)	4	3.47 (± 0.61)	4	3.53 (± 0.64)	4	0.603
9. Influenza is an overvalued disease *	1.38 (± 0.50)	1	2.46 (± 0.95)	2	2.73 (± 0.80)	3	<0.001
10. My contacts expect me to be up to date on vaccinations *	3.33 (± 0.73)	3	2.71 (± 0.93)	3	2.92 (± 0.73)	3	0.021
11. I constantly recommend the flu shot to my colleagues *	2.9 (± 0.96)	3	1.88 (± 1.04)	2	1.46 (± 0.92)	1	<0.001
12. I constantly recommend the flu shot for those who are diabetic and have heart disease or renal failure *	3.8 (± 0.51)	4	3.02 (± 0.92)	3	2.53 (± 1.25)	2	<0.001
13. Since the mass media talk about adverse vaccine reactions, I trust vaccines less than before *	1.09 (± 0.30)	1	1.52 (± 0.82)	1	2 (± 1.13)	2	0.008
14. Some of the side effects of vaccines are kept hidden from us *	1.14 (± 0.36)	1	1.6 (± 1.41)	1	2.66 (± 1.29)	3	<0.001
15. Vaccine excipients have serious side effects	1.47 (± 0.60)	1	1.82 (± 0.77)	2	2 (± 0.82)	2	0.087
16. There should be more mandatory vaccines for health professionals *	3.33 (± 1.06)	4	2.34 (± 1.07)	2	2.46 (± 0.97)	3	0.001
17. There should be more mandatory vaccines for those who work with the public *	3.38 (± 1.02)	4	2.36 (± 1.04)	2	2.53 (± 1.05)	3	0.001
18. I believe that the health surveillance of adverse vaccine reactions is ineffective *	1.57 (± 0.68)	2	2.15 (± 0.92)	2	2 (± 0.91)	2	0.034
19. Everyone should have the right to choose whether or not to get vaccinated *	1.42 (± 0.81)	1	2.15 (± 1.12)	2	2.76 (± 1.17)	3	0.002

* *p* < 0.05

**Table 7:** Distribution of healthcare worker’s vaccination compliance (HVCI) scores, with the hypothesis of the study and related p-value. ICU: Intensive care unit; MAN: management; DIAG: diagnostics; EMERG: emergency room; ONC-HEM: oncohematology. PHIS: physicians; NUR: nurses; AUX: auxiliary staff. YFLU: flu shot has occurred; NFLU: flu shot has not occurred; SKILLS: high skills; NO SKILLS: low skills; INGROUP 1: influential ingroup; INGROUP 2: non-influential ingroup.

HP1							
	MIN	1°QUART	MEDIAN	MEAN	3°rd QUART	MAX	*p* VALUE
ICU	0.64	1.30	1.75	1.88	2.29	4.00	*p* = 0.03 *
ONC-HEM	1.00	1.28	1.87	2.06	2.83	4.00	
EMERG	0.46	1.02	2.00	1.95	2.58	3.80	
DIAG	0.57	2.09	1.39	2.71	3.60	4.00	
MAN	1.30	1.85	2.86	2.61	3.30	3.80	
HP2							
	MIN	1°QUART	MEDIAN	MEAN	3°rd QUART	MAX	*p* VALUE
PHIS	2.17	2.57	2.83	2.99	3.60	4.00	*p* < 0.01 *
NUR	0.46	1.27	1.86	1.87	2.43	4.00	
AUX	0.57	0.95	1.36	1.88	1.96	2.83	
HP3							
	MIN	1°QUART	MEDIAN	MEAN	3°rd QUART	MAX	*p* VALUE
YFLU	1.67	1.88	2.33	2.39	2.70	3.80	*p* < 0.01 *
NFLU	0.69	1.18	1.71	1.91	2.33	3.40	
HP4							
	MIN	1°QUART	MEDIAN	MEAN	3°rd QUART	MAX	*p* VALUE
HVCI SKILLS	0.69	1.50	2.22	2.13	2.57	3.80	*p* = 0.04 *
HVCI NO SKILLS	0.64	1.18	1.86	1.95	2.33	3.40	
HP5							
	MIN	1°QUART	MEDIAN	MEAN	3°rd QUART	MAX	*p* VALUE
HVCI INGROUP1	0.91	1.27	1.87	2.27	3.25	4.00	*p* = 0.07
HVCI INGROUP2	0.46	1.55	2.22	2.17	2.80	4.00	

* *p* < 0.05

**Table 8:** Results from the multivariate logistic regression analysis. The outcome variable is the flu shot received in the epidemic season of 2017–2018. The independent variables are sex, self-perceived skills, and HVCI.

Variables		OR	[95% CI]	*p*
Sex *	Males	1	-	-
	Females	0.119	[0.027–0.518]	0.005
Skills	Excellent	1	-	-
	Good	0.054	[0.002–1.794]	0.103
	Discrete	0.0197	[0.038–1.018]	0.053
	Poor	0.05	[0.007–0.338]	0.002
HVCI°		3.570	[1.703–7.484]	0.001

°HVCI was considered as a continuous variable; * *p* < 0.05
